# Self‐Assembling Peptide‐Based Hydrogels for Wound Tissue Repair

**DOI:** 10.1002/advs.202104165

**Published:** 2022-02-09

**Authors:** Tong Guan, Jiayang Li, Chunying Chen, Ying Liu

**Affiliations:** ^1^ CAS Key Laboratory for Biomedical Effects of Nanomaterials and Nanosafety & CAS Center for Excellence in Nanoscience National Center for Nanoscience and Technology of China Beijing 100190 P. R. China; ^2^ University of Chinese Academy of Sciences Beijing 100049 P. R. China; ^3^ GBA National Institute for Nanotechnology Innovation Guangdong 510700 P. R. China

**Keywords:** hydrogels, microenvironment regulation, self‐assembling peptides, spatiotemporal control, wound tissue repair

## Abstract

Wound healing is a long‐term, multistage biological process that includes hemostasis, inflammation, proliferation, and tissue remodeling and requires intelligent designs to provide comprehensive and convenient treatment. The complexity of wounds has led to a lack of adequate wound treatment materials, which must systematically regulate unique wound microenvironments. Hydrogels have significant advantages in wound treatment due to their ability to provide spatiotemporal control over the wound healing process. Self‐assembling peptide‐based hydrogels are particularly attractive due to their innate biocompatibility and biodegradability along with additional advantages including ligand‐receptor recognition, stimulus‐responsive self‐assembly, and the ability to mimic the extracellular matrix. The ability of peptide‐based materials to self‐assemble in response to the physiological environment, resulting in functionalized microscopic structures, makes them conducive to wound treatment. This review introduces several self‐assembling peptide‐based systems with various advantages and emphasizes recent advances in self‐assembling peptide‐based hydrogels that allow for precise control during different stages of wound healing. Moreover, the development of multifunctional self‐assembling peptide‐based hydrogels that can regulate and remodel the wound immune microenvironment in wound therapy with spatiotemporal control has also been summarized. Overall, this review sheds light on the future clinical and practical applications of self‐assembling peptide‐based hydrogels.

## Graphical abstract

The main self‐assembling peptide‐based systems, the advantages of self‐assembling peptide‐based hydrogels, and the rational design of peptide‐based materials for different stages of wound healing are reviewed. Advanced self‐assembling peptide‐based materials that can be applied in spatiotemporally controllable, multifunctional wound healing and regulate/remodel the wound microenvironment are discussed. Prospects and challenges related to peptide‐based hydrogels for wound healing are also highlighted.

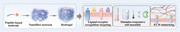

## 1 Introduction

Wounds produced by trauma, burns, surgery, and chronic diseases cause many millions of patients to suffer discomfort, pain, and, in severe cases, disability.^[^ ^1^ ^]^ The burden of chronic wounds is growing due to the increasing incidence of obesity and diabetes, resulting in therapeutic problems and increased medical costs.^[^ ^2^ ^]^ Therefore, simple and effective wound treatment remains a difficult problem that presents substantial challenges to the medical system.

Wound tissue repair, particularly chronic wound healing, is a complicated and multistage biological process. At present, a variety of treatment types are available for wounds.^[^ ^3^ ^]^ Invasive surgical treatments (e.g., surgical debridement and skin grafting) are associated with risk of bleeding or tissue damage, require bed rest and recovery, and result in high medical costs. Gel dressings have shown great potential in non‐invasive therapy due to the following advantages: A 3D structure resembling soft tissue; excellent in situ solid gel formation after injection; and the ability to serve as targeted delivery vehicles of drugs, proteins, or cells.^[^ ^4^ ^]^ The global market sales and demand for medical dressings are growing every year. Compared with low‐end dressings, high‐end wound dressings are more in demand and have broader market prospects. Thus, different classes of biomaterial‐based hydrogel dressings have been developed for wound healing, including dressings based on peptides, chitosan, collagen, alginate, heparin, cellulose, and hyaluronic acid (**Table** **1**).

**Table 1:** Various developed gel dressings and their applications

Dressing type	Characteristics	Application and details	Ref.
Chitosan	3D polymer network, strong water absorption, excellent compatibility, non‐adhesive, degradable, and cost‐effective.	For acute to chronic wounds, exudating, contaminated wounds, venous leg ulcers, diabetes, and first‐ and second‐degree burns. Have hemostatic and bacteriostatic action, and accelerate healing processes.	[150]
Collagen and gelatin	Thermally and chemically stable with high tensile strength, permeable to O_2_, highly biocompatible, unable to retain, and expensive.	Cover burn wounds and treat ulcers. Resistant to bacterial attack as well as further mechanical trauma.	[151]
Alginate	Fibrous, highly absorbent, need a second dressing layer to avoid drying.	Treat infected and noninfected wounds, and inappropriate for dry wounds.	[152]
Heparin	Efficient binding with a variety of growth factors.	Treat burns and diabetic foot ulcers. Participate in wound angiogenesis, cell growth, migration, and differentiation.	[153]
Cellulose	3D network structure, high tensile strength, strong water holding capacity, permeability to gas and liquid.	Used in scalds and ulceration. Facilitate autolytic debridement, improve the development of granulation tissue, and accelerate re‐epithelialization.	[154]
Hyaluronic acid	ECM component, high water absorption and permeability, and fast degradation.	Heal burns, epithelial surgical, and chronic wounds. Modulate wound via specific HA receptors, inflammation, cellular migration, and angiogenesis.	[155]

Peptide‐based wound dressings have received extensive attention. Peptides exist widely in the human body and have specific biological functionality, activity, and specificity; thus, natural and artificially synthesized peptide molecules exhibit a wide range of biological effects.^[^ ^5^ ^]^ For example, a variety of peptides (e.g., OA‐GL12, OA‐GL21, RL‐QN15, and Ot‐WHP) derived from the skin secretions of amphibians accelerate wound healing (including acute wounds and diabetic chronic wounds).^[^ ^6^ ^]^ Moreover, some peptide growth factors such as, epidermal growth factor (EGF) and fibroblast growth factor (FGF) promote wound healing by playing essential roles in wound proliferation and tissue remodeling.^[^ ^7^ ^]^

Some peptide sequences can self‐assemble into supramolecular structures through weak intramolecular or intermolecular interactions. Reasonably designed self‐assembling peptides have a vast array of functions, including mimicking the extracellular matrix (ECM),^[^ ^8^ ^]^ activating humoral and cellular immunity,^[^ ^9^ ^]^ and assisting in drug delivery and targeting.^[^ ^10^ ^]^ Additionally, low‐molecular‐weight peptide‐based hydrogelators offer better biocompatibility and biodegradability than common polymer gels.^[^ ^11^ ^]^ Thus, these hydrogelators rarely induce severe adverse effects and meet the requirements for most tissue engineering applications. As a result, self‐assembling peptide‐based hydrogels have comprehensive applications in nanotechnology and biomedicine, including topical drug delivery, tumor treatment, immune adjuvant, 3D tissue cell culture, tissue repair, and tissue regeneration.^[^ ^10^, ^12^ ^]^

Self‐assembling peptide‐based molecules have also shown considerable potential for multimodal treatment due to their ability to respond to changes during complicated wound healing processes. Self‐assembling peptides with ordered structures can be manufactured by encoding peptide sequences to obtain various microscopic structures, including spheres, vesicles, micelles, nanofibers, and nanotubes.^[^ ^13^ ^]^ These microscopic structures further endow the self‐assembling peptides with additional properties; for example, reported structures include nanospheres that encapsulate and deliver drugs and nanofibers that can entangle aqueous media into hydrogels. Moreover, the peptide‐based fibrous network is similar in structure and composition to fibrin in the ECM, which facilitates the repair of damaged tissues and the restoration of their biological functions. The rational incorporation of functional motifs into self‐assembling peptide building blocks makes peptide‐based hydrogels attractive as functional biomaterials.^[^ ^14^ ^]^ Peptide‐based hydrogels can be controllably self‐assembled under the influence of temperature, pH, ions, and enzymes to better fit the edges of wounds compared to other wound dressings. Furthermore, the forming hydrogels can be pre‐seeded with cells and functional molecules, and the constituents of the hydrogels can be modified to achieve multiple functions that promote wound healing.^[^ ^3a^ ^]^ Consequently, exploiting the properties of peptide‐based hydrogels can make it easier to obtain a molecular design that facilitates wound healing.

**Figure 1:**
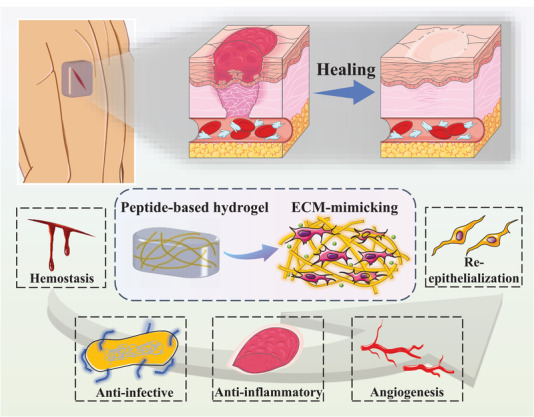
Schematic illustration of the influence of peptide‐based self‐assembling hydrogels on the wound healing process. Part of the figure is modified from Servier Medical Art (http://smart.servier.com/), licensed under a Creative Common Attribution 3.0 Generic License.

Although various reviews have focused on wound healing, there is a lack of comprehensive reviews focusing on the application of self‐assembling peptide‐based hydrogels in wound healing. This review provides a comprehensive summary of current peptide‐based self‐assembling hydrogel systems used in wound healing (**Figure** **1**). We have also discussed the roles and mechanisms of self‐assembling peptide‐based hydrogels in various wound healing processes, including hemostasis, infection and inflammation response, proliferation, and tissue remodeling. This review introduces several types of self‐assembling peptide‐based materials and their unique advantages that show promise for wound treatment with spatiotemporal control. The personalization of self‐assembling peptide‐based hydrogel dressings will result in more clinical and translational potential in wound treatment.

## 2 Wound Healing

Wound healing is a complicated and well‐orchestrated process that starts immediately after injury. The personalized design of hydrogel dressings to provide advanced spatiotemporal control over wound treatment, including hemostasis, infection and inflammation response, angiogenesis, and re‐epithelialization, is urgently needed.

### 2.1 Progression and Characteristics of Wound Healing

#### 2.1.1 Normal Wound Repair

**Figure 2:**
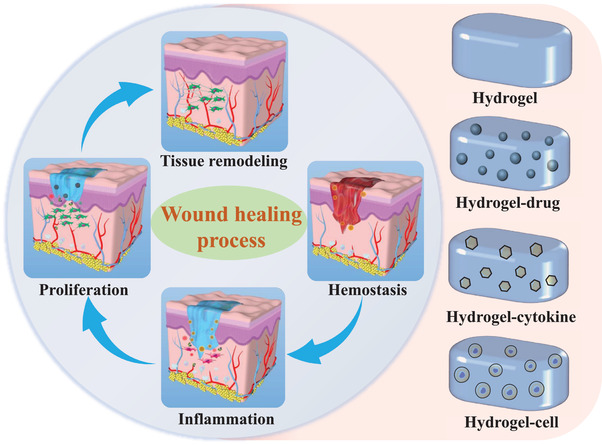
Hydrogel systems used in the four typical stages of wound healing. The schematic diagram of the wound healing process is redrawn from Ref. [15e]. Reproduced with permission.^[^ ^15e^ ^]^ Copyright 2020, American Chemical Society.

The process of normal mammalian dermal wound repair is divided into four overlapping but distinct stages: a) Hemostasis and early inflammatory response; b) late inflammatory response (removing dead and devitalized tissues and preventing infection); c) angiogenesis and new tissue proliferation; and d) tissue remodeling (**Figure** **2**).^[^ ^15^ ^]^ The entire process is tightly regulated by multiple cells, and numerous growth factors, cytokines, and chemokines are secreted to achieve barrier closure and functional recovery.^[^ ^16^ ^]^

When dermal injury occurs, the first stage in healing is to stop the bleeding and minimize hemorrhage, which is a rapid process achieved through the contraction of blood vessels, platelet aggregation, and blood clot formation.^[^ ^15a^ ^]^ In the next stage, inflammation occurs immediately after tissue damage. Inflammation lasts longer in chronic and infected wounds. Several inflammatory cells and cytokines are recruited and activated during homeostasis.^[^ ^17^ ^]^ These cytokines also promote the migration of fibroblasts, leading to the start of the next stage: Tissue proliferation.^[^ ^15b^ ^]^ At this stage, new blood vessels form (i.e., angiogenesis or neovascularization) at the wound, and ECM proteins (e.g., collagen fibers and granulation tissues) are synthesized and deposited,^[^ ^18^ ^]^ forming a new substrate for keratinocyte migration in the next phase. Vascular endothelial growth factor A (VEGFA) plays an essential role in the regulation of angiogenesis.^[^ ^15a^ ^]^ The final stage is organizational remodeling, which lasts a long time. During this phase, most activated inflammatory cells undergo programmed cell death or outflow from the wound, mainly leaving ECM proteins and collagen. Collagen is remodeled and scarred, marking the completion of the wound healing process. Pathogenic infection will slow the rate of wound healing and can be life‐threatening in severe cases.^[^ ^19^ ^]^ Therefore, it is critical to prevent and treat infections during wound healing.

#### 2.1.2 Chronic Wound Repair

The majority of chronic wounds fall under three categories: Vascular ulcers, diabetic ulcers, and pressure ulcers. The pathophysiological phenomena associated with chronic wounds include prolonged or excessive inflammation, persistent infection, impaired angiogenesis, difficult re‐epithelialization, dysregulated levels of cytokines/growth factors, and increased protease activity.^[^ ^20^ ^]^

The levels of pro‐inflammatory cytokines (IL‐1, IL‐6, and TNF‐*α*) and inflammatory chemokines (MCP‐1 and IL‐8) in chronic wounds are higher than those in acute wounds; this leads to the increased production of matrix metalloproteinases (MMPs) and reductions in the tissue inhibitors of MMPs, thereby inhibiting ECM formation and dermis reconstruction.^[^ ^21^ ^]^ Chronic wounds are also associated with hyperproliferative keratinocytes, the overexpression of the proliferation marker Ki67,^[^ ^21a^ ^]^ impaired migration, and deficient expressions of differentiation markers. Due to these abnormalities, chronic wound closure is difficult. Chronic wounds are clinically challenging to treat and bear high health and cost burdens. The transition from the inflammatory stage to the proliferative stage represents a key step during chronic wound healing;^[^ ^22^ ^]^ developing strategies to effectively and quickly facilitate this transition is crucial for enhancing the efficacy of chronic wound therapy.

### 2.2 Hydrogel Systems for Wounds

The earliest treatment for wounds was the application of natural or synthetic bandages, often cotton or gauze, to keep the wound dry and prevent bacteria from entering.^[^ ^23^ ^]^ Subsequent studies demonstrated that a warm and moist environment can lead to faster and more successful wound healing.^[^ ^23^ ^]^ The use of hydrogel formulations can achieve wound healing with spatiotemporal control and is thus considered an effective strategy.^[^ ^24^ ^]^ Therefore, a series of hydrogel dressings have been developed for treating wounds.

As depicted in Figure 2, the hydrogel systems currently used for wound treatment include hydrogel, hydrogel–drug, hydrogel–cytokine, and hydrogel–cell systems.^[^ ^25^ ^]^ Compared with other materials, hydrogels and particularly peptidyl hydrogels have some advantages in terms of their in vivo and environmental toxicity. Macroscopically, hydrogels have high water content, tunable mechanical stability, excellent injectability, and outstanding biocompatibility.^[^ ^5a^ ^]^ Microscopically, reasonably designed peptide sequences can not only target specific receptors, they can also respond to various in situ stimuli to self‐assemble. With the rational regulation of the self‐assembly process, peptide‐based self‐assembling hydrogels can afford functionalized microscopic structures that act as a provisional ECM to facilitate the migration and proliferation of various cells involved in wound healing and promote the microenvironmental remodeling of physiological tissues. These characteristics make peptide‐based self‐assembling hydrogels popular and valuable in biomedical applications.

## 3 Self‐Assembling Peptides

### 3.1 Self‐Assembly

Self‐assembly, which refers to the spontaneous organization of molecules into more prominent and structured arrangements, is a miraculous and common phenomenon in nature and in organisms. Self‐assembly phenomena occur across a variety of scales from tiny cells to the sizeable human body. Importantly, self‐assembly processes are critical for the “bottom‐up” construction of nanostructures,^[^ ^26^ ^]^ primarily driven by thermodynamics. However, kinetics are also a critical factor in structural modulation and functional integration.^[^ ^27^ ^]^

**Figure 3:**
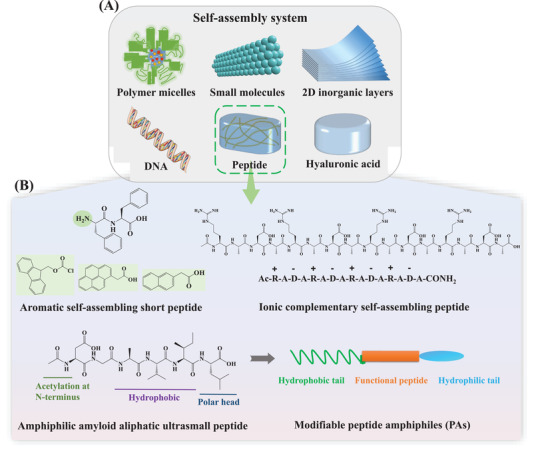
Overview of the self‐assembly of peptides. A) Schematic illustration of extensively researched self‐assembly systems. B) The four main peptide self‐assembly systems and representative examples: Aromatic self‐assembling peptides (FF and common N‐terminal aromatic substituent groups: naphthalene, Fmoc, and pyrene); ionic complementary self‐assembling peptides (RADA16); amyloid aliphatic self‐assembling ultrasmall peptides (Ac‐LD_6_: Ac‐LIVAGD); and modifiable peptide amphiphiles (PAs). (Fmoc: Fluorenylmethyloxycarbonyl).

The development of medicines is becoming more and more predictive, personalized, and regenerative, and the precise structural and functional control of nanomaterials at the molecular level is required to create customized nanostructures. Self‐assembly can meet these demands by allowing the synthesis of functional nanomaterials from precisely designed building blocks. The resulting nanomaterials have comprehensive applications in nanotechnology and biomedicine, including molecular imaging,^[^ ^28^ ^]^ cancer therapy,^[^ ^29^ ^]^ and tissue engineering.^[^ ^30^ ^]^ Therefore, a wide range of materials have been extensively studied for the preparation of self‐assembling nanomaterials, including polymer micelles,^[^ ^31^ ^]^ small molecules,^[^ ^32^ ^]^ 2D inorganic layers,^[^ ^33^ ^]^ DNA,^[^ ^34^ ^]^ hyaluronic acid derivatives,^[^ ^35^ ^]^ and peptides (**Figure** **3**A).

### 3.2 Peptide‐Based Self‐Assembly Systems

At present, molecular self‐assembly is regarded as a valuable approach for producing novel materials. Peptides are among the most popular building blocks and programmable molecular motifs for self‐assembly. Peptides have recognizable characteristics with a variety of sequences and structures, promising them critical signaling molecules in biological systems. Self‐assembly can be used as a fabrication strategy in peptides to create various architectures from nanotubes on the nanoscale to fiber bundles on the macroscale with specific conformations (e.g., *β*‐sheet and *α*‐helix). Peptide nanofibers can also self‐assemble through bundling and entangling to form 3D networks under physiological conditions.

The conjugated self‐assembling nanostructures of peptides are generally formed through non‐covalent interactions, including *π*–*π* interactions, charge‐based interactions, hydrophobic interactions, hydrogen bond interactions, and van der Waals forces.^[^ ^36^ ^]^ These non‐covalent interactions can enhance the mechanical properties of hydrogel elastomers and improve their self‐healing ability.^[^ ^37^ ^]^ Moreover, non‐covalent interactions are critical for forming stable hydrogels and should thus be considered when designing peptide‐derived gelators. In addition to the hydrogen bonds between amide bonds, self‐assembling peptide systems have been developed based on various other interactions (Figure 3B).

#### 3.2.1 Aromatic Self‐Assembling Short Peptide Systems

*π*–*π* interactions are one of the critical interactions for the construction of peptide‐based nanomaterials.^[^ ^38^ ^]^ Diphenylalanine (FF) is the simplest natural self‐assembling peptide that has been reported.^[^ ^39^ ^]^ Based on *π*–*π* interactions, FF can further stabilize the formed *β*‐sheet structure.^[^ ^38^ ^]^ The addition of a chemically modified aromatic group at the N‐terminal end further strengthens the *π*–*π* interactions between molecules and promotes the formation of more stable hydrogels.^[^ ^38^, ^40^ ^]^ For example, when FF is modified with fluorenylmethyloxycarbonyl (Fmoc) at the N‐terminal end (Fmoc‐FF), self‐assembly still occurs.^[^ ^41^ ^]^ Moreover, the secondary structure of Fmoc‐FF nanofibers undergoes a charge‐induced transformation from *β*‐sheet to *α*‐helix.^[^ ^42^ ^]^ The simple water‐soluble precursor, Fmoc‐tyrosine (Fmoc‐Yp), dephosphorylated by alkaline phosphatase (ALP), can also self‐assemble into a nanofibrillar hydrogel network.^[^ ^43^ ^]^ Xu and co‐workers found that using naphthalene (Nap) instead of Fmoc to connect FF (Nap‐FF) resulted in stronger *π*–*π* interactions, leading to the preparation of a series of enzymatic self‐assembly systems.^[^ ^44^ ^]^

#### 3.2.2 Ionic Complementary Peptide Systems

Non‐aromatic ionic self‐complementary oligopeptides mainly form hydrogels through electrostatic interactions. Ionic complementary peptides are characterized by the alternate arrangement of positive and negative charges. The charge distribution plays a major role in the self‐assembly of these peptides. Three charge distribution patterns are widely used in the design of self‐complementary peptides: type I, − +; type II, −− ++; and type III, −−−− ++++,^[^ ^45^ ^]^ for example, EAK16 and RADA16. One side of RADA consists of nonpolar hydrophobic alanine (A), while the other side consists of alternating oppositely charged amino acids [positively charged arginine (R) and negatively charged aspartic acid (D)].^[^ ^46^ ^]^ EAK16 and RADA16 show good self‐assembly to form stable *β*‐sheet layers in aqueous solution,^[^ ^46^ ^]^ and they are becoming more and more important in biomedicine.^[^ ^12^, ^47^ ^]^

#### 3.2.3 Amphiphilic Amyloid Aliphatic Ultrasmall Peptide Systems

In addition to the above two kinds of non‐covalent interactions, rationally designed peptides can also assemble through hydrophilic and hydrophobic interactions. Amyloid fiber aggregates represent a class of short aliphatic tri‐ to hexa‐peptides of a specific sequence. As the simplest and smallest non‐aromatic structures, they can self‐assemble into stable helical fibrous aggregates in water. Their peptide motif contains a tail of aliphatic nonpolar amino acids at the N terminus with reduced hydrophobicity and a hydrophilic head group of basic, neutral, or acidic nonaromatic polar amino acids at the C terminus.^[^ ^48^ ^]^ Among them, hexamers (Ac‐LIVAGD) are most likely to form hydrogels.^[^ ^48^ ^]^ Polypeptides self‐assemble into amyloid *β*‐type fiber aggregates through intermediate *α*‐helical structures; this process includes multiple steps, the key of which is the transition from a random coil structure to an *α*‐helical conformation.^[^ ^48^ ^]^ This type of hydrogel has high water content and excellent biocompatibility in cells, making it an attractive biological material.^[^ ^49^ ^]^

#### 3.2.4 Modifiable Peptide Amphiphile Systems

In addition to amphipathic aliphatic ultrashort peptides with specific sequences, Stupp and co‐workers have designed and synthesized a broad range of self‐assembling peptide amphiphiles (PAs), which combine the structural features of amphiphilic surfactants with the functions of bioactive peptides.^[^ ^50^ ^]^ PA molecules are typically formed from a hydrophobic block (in most cases, a long alkyl chain) and a short peptide sequence capable of forming intermolecular hydrogen bonds and usually *β*‐sheet cylindrical nanofibers.^[^ ^51^ ^]^ In aqueous solution, the hydrophobic tail can act as a shield against water, induce and stabilize the 3D structure of the peptide group, and drive the self‐assembly process. Moreover, PAs include charged amino acids following the hydrophobic peptide sequence, leading to enhanced solubility in water. Specific peptide can be combined at the end of the molecule to obtain a biologically active structure.^[^ ^52^ ^]^ Compared with the peptides mentioned in the preceding sections, PAs are more dependent on the amphiphilicity of the chemical structure and less dependent on the peptide sequence, which expands the range of possible peptides.

In summary, the self‐assembly process shows promise for producing customized nanoscale biomaterials for various medical applications. For example, self‐assembling peptide‐based materials have been used extensively in anticancer therapy,^[^ ^53^ ^]^ antimicrobials,^[^ ^54^ ^]^ and vaccines.^[^ ^55^ ^]^ Self‐assembling peptide‐based materials also show great potential for the controlled release of bioactive molecules,^[^ ^56^ ^]^ imaging‐based diagnosis,^[^ ^38^ ^]^ and regenerative medicine.^[^ ^51^ ^]^

## 4 Characteristics of Peptide‐Based Self‐Assembling Hydrogels

**Figure 4:**
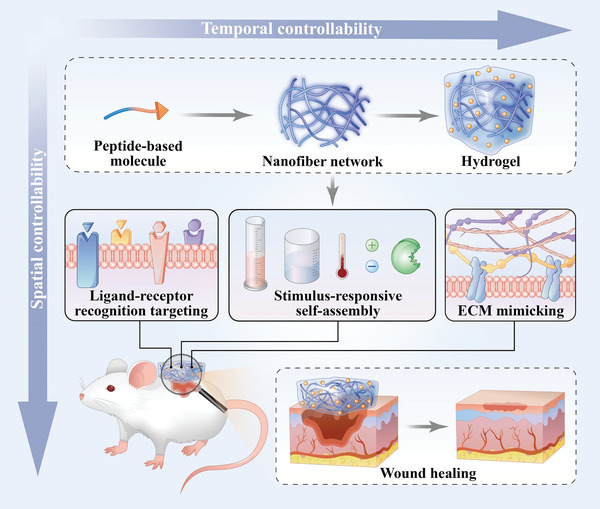
Advantages of peptide‐based self‐assembling hydrogels. Peptide‐based hydrogels provide spatiotemporal control over the wound healing process. In terms of temporal control, peptide‐based hydrogels can achieve long‐term wound treatment, sustained drug release, and controlled degradation. In terms of spatial control, the soft hydrogels can fit the edges of wound tissue, the nanofibrous structure can mimic the extracellular matrix, and the scaffold can regulate the wound microenvironment.

Due to the outstanding characteristics of peptide‐based self‐assembling hydrogels in the field of biomedicine, they can serve multiple functions to promote wound healing and allow spatiotemporal control over the complicated wound healing process (**Figure** **4**). This section describes some critical biological functions of peptide‐based assemblies, including ligand‐receptor recognition, stimulus‐responsive self‐assembly, and ECM mimicking. These characteristics give self‐assembling peptide‐based materials better therapeutic potential in wound healing compared to other materials.

### 4.1 Ligand‐Receptor Recognition

Short peptides have been widely used as targeted ligands to guide drug delivery based on ligand‐receptor‐specific binding.^[^ ^57^ ^]^ The addition of peptide ligands to other biologically inert substrates allows desired tissues or receptors to be targeted. To achieve the targeted treatment of wounds, peptide sequences can be designed according to the features of tissues or cells.

For instance, RGD (Arg‐Gly‐Asp), a bioactive cellular adhesion ligand, has been extensively used to accelerate the process of wound healing because it can target integrin receptors on the cell membrane, promote cell migration, and accelerate the formation of granulation tissue.^[^ ^58^ ^]^ Moreover, several active peptides including CAG, REDV, and YIGSR can be recognized explicitly by endothelial cells (ECs). Therefore, these peptides can be used to modify materials to achieve the selective adhesion of ECs. At the same time, these active peptides stimulate the proliferation of ECs to facilitate blood vessel formation during wound treatment.^[^ ^59^ ^]^ In addition, relatively short cell‐penetrating peptides with positive net charges (e.g., sC18) can be coupled with Tylotoin, a wound healing peptide, to improve the delivery of Tylotoin across cellular membranes and entry into keratinocytes.^[^ ^60^ ^]^ The internalized peptide further promotes the proliferation of keratinocytes, which is a critical step in wound healing.^[^ ^60^ ^]^

### 4.2 Stimulus‐Responsive Self‐Assembly

**Figure 5:**
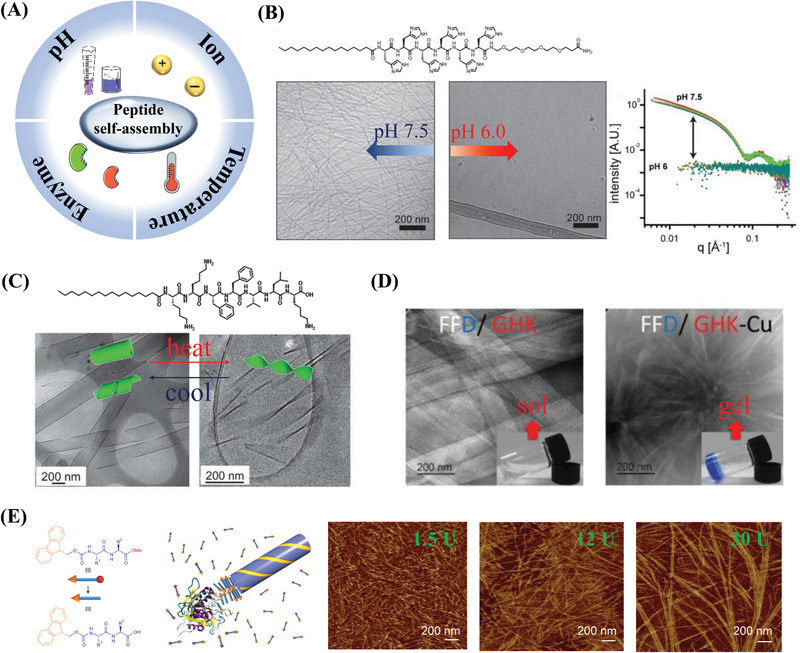
Stimuli‐responsive self‐assembly of peptides. A) Schematic illustration of peptide‐based self‐assembly in response to pH, temperature, ions, and enzymes. Part of the figure is modified from Servier Medical Art (http://smart.servier.com/), licensed under a Creative Common Attribution 3.0 Generic License. B) Changes in pH trigger the reversible assembly and disassembly of C_16_H_6_‐OEG nanofibers. Cryogenic transmission electron microscopy (cryo‐TEM) images and small‐angle X‐ray scattering patterns demonstrate that PA assembles into nanofibers at pH 7.5 and disassembles at pH 6.0, and this process is reversible. Reproduced with permission.^[^ ^62^ ^]^ Copyright 2014, American Chemical Society. C) Temperature‐driven reversible helix formation and unwinding of a self‐assembling structure (C_16_‐KKFFVLK). Cryo‐TEM images show an unwinding transition leading to twisted tapes upon heating, while nanotubes and ribbons re‐form upon cooling. Reproduced with permission.^[^ ^66^ ^]^ Copyright 2013, Royal Society of Chemistry. D) Copper ions trigger peptides (FFD/GHK) to form a long‐fiber hydrogel. TEM images show that the presence of Cu^2+^ facilitates the assembly of the tripeptides, resulting in hydrogelation. Reproduced with permission.^[^ ^69^ ^]^ Copyright 2017, Royal Society of Chemistry. E) Enzymatic hydrolysis promotes the self‐assembly, nucleation, and structural growth of Fmoc‐dipeptide methyl esters. Atomic force microscopy (AFM) images corresponding to different enzyme concentrations suggest that increasing the enzyme concentration promotes the *π*–*π* interactions and leads to a more ordered fiber supramolecular structure. Reproduced with permission.^[^ ^77^ ^]^ Copyright 2010, Springer Nature.

Based on the relatively weak assembly interactions in peptide‐based materials, various physiological parameters such as, pH, temperature, ions, and enzymes can be used to precisely modulate the non‐covalent interactions and regulate the assembly and performance of the peptides (**Figure** **5**A and **Table** **2**). A reasonably designed peptide can undergo a sol‐to‐gel transition in response to physiological stimuli, facilitating rapid hemostasis and wound filling (in irregular or deep wounds) upon injection. Moreover, the controllable release of drugs for wound treatment can also be achieved using stimuli‐responsive materials.

**Table 2:** Stimulus‐responsive peptide‐based self‐assemblies

Physiological stimulus	Peptide sequence	Features	Application	Ref.
	GALA (WEAALAEALAEALAEHLAEALAEALEALAA‐amide)	GALA interacts and destabilizes the lipid bilayers at acidic pH.	Drug, gene delivery, and cancer therapy	[156]
	pHMAPS (PpIX‐AEQNPIYWARYADWLFTTPLLLLDLALLVDADEGT)	Tumor acidic pH triggers a conformation switch of pHMAPS for inserting into tumor cell membranes.	Anti‐tumor growth and metastasis	[157]
pH	PA: C_16_‐KTTKS	As the pH decreases, the morphology of the material changes from tapes to twisted brils, to tapes, to micelles.		[61]
	PA: C_16_H_6_‐OEG	pH controls the reversible assembly and disassembly of nanofibers and spherical micelles.	Drug encapsulation and tumor accumulation	[62]
	PA: C16‐CCCCGGGS(P)‐RGD	At pH4, PA forms a long nanofibrous scaffold and disassembles when pH increases.		[52a]
	Cationic peptide: I_6_K_5_	The destruction of the nanoparticles occurred under acidic pH conditions.	Drug release	[63]
	PA: C_16_‐V_3_A_3_K_3_	Temperature affects the length of nanofibers.		[64]
Temperature	PA: C_16_‐VVVAAAKKK	The increase in temperature leads to an increase in the proportion of *β*‐sheet structures.		[65]
	PA: C_16_‐KKFFVLK	Temperature changes cause reversible thermal transitions between nanotubes and helical ribbons.		[66]
	Fmoc‐KCRGDK	The increase in temperature causes self‐assembled micelles to transform into assembled nanofibers.	Drug encapsulation for tumor immunotherapy	[158]
	KLVFFAK	Ionic strength tunes the size and yield of an amyloid‐like nanosheet.	Retroviral gene transduction	[68]
	FFD/GHK	Copper(II) ions promote the formation of nanofibrous hydrogels.		[69]
	GHK	GHK has a high affinity for copper(II) ions and spontaneously forms a tripeptide–copper complex (GHK‐Cu).	Wound healing	[71]
Ion	FF8 (KRRFFRRK)	Negatively charged lipid membranes induce self‐assembly of FF8.	Antibacterial	[72]
	Naproxen‐FF	Metal ions modulate the self‐assembly process and the mechanical properties of the hydrogel.		[114c]
	APAs: K_S_XEK_S_ (X = variable amino acid residue)	The increase in ion concentration results in a transition from flat nanoribbons to twisting nanohelices.		[159]
	A series of seven‐residue peptides	Peptides can self‐assemble in the presence of transition‐metal ions to form catalytic amyloids.	Promoting hydrolytic and redox transformations	[160]
	Fmoc‐Y_P_	ALP converts precursor to hydrogelator by dephosphorylation and then promotes self‐assembling.		[43]
	Nap‐_D_‐Phe‐_D_‐Phe‐_D_‐Tyr(H_2_PO_3_)		Cancer cell killing	[161]
	IR775‐Phe‐Phe‐Tyr(H_2_PO_3_)‐OH		Tumor PA imaging	[162]
	Nap‐FFGEY	Kinase and phosphatase regulate the formation/dissociation of self‐assembling nanostructures.		[78]
Enzyme	Fmoc‐dipeptide methyl esters	Subtilisin hydrolyzes methyl ester groups to promote self‐assembling.		[77]
	Nap‐phe‐phe‐NHCH_2_CH_2_OH	Esterase hydrolyzes ester bonds to promote the self‐assembly of the hydrogelator.	Cancer cell killing	[75]
	PhAc‐FFAGLDD	MMP‐9 digestion promotes the peptide structure from micellar aggregates to fibers.	Drug encapsulation, release, and cancer therapy	[76a,b]
	PA: C_12_‐GGRGDRPLGVRVVV	MMP‐2 digestion degrades peptide filaments and reassembles them into spherical micelles.	Drug release and tumor cell killing	[53]
	Olsa‐RVRR	Furin digestion and GSH reduction initiate a condensation reaction to promote the self‐assembly of nanostructures.	Tumor imaging and therapy	[163]

#### 4.2.1 Responsiveness to pH

The pH is an easily controlled and widely studied stimulus. Among the amino acid residues in peptide sequences, pH mainly affects the amino and carboxyl groups. Charged amino acids are key in designing pH‐responsive self‐assembling molecules. The pH of the solution affects the charges and other properties of peptide self‐assembling materials by modulating the competitive solvation between the donor and acceptor sites of the hydrogen bonds. For example, as the pH of the solution increases, the structure of PA often changes from spherical micelles to nanofibers.^[^ ^61^ ^]^ A peptide‐based amphiphilic molecule containing oligo‐histidine H_6_ can reversibly self‐assemble in response to pH changes. It self‐assembles into nanofibers at pH 7.5 and disassembles into spherical shape at pH 6.0 (Figure 5B).^[^ ^62^ ^]^ Moreover, different peptide‐based sequences will have different responses to pH due to differences in charge distribution. The pH‐triggered self‐assembly of PA formed a long nanofibrous scaffold at pH 4 that disassembled when the pH increased.^[^ ^52a^ ^]^ In addition to PA, pH also triggers changes in the geometry of cationic peptides (I_6_K_5_). In weakly acidic environments, many random coil structures form, leading to nanoparticle (NPs) destruction.^[^ ^63^ ^]^ This stimulus‐triggered self‐assembly process has a wide range of applications in cell uptake and drug delivery and also provides inspiration for the preparation of physiological pH‐responsive wound dressings.

#### 4.2.2 Responsiveness to Temperature

Temperature is an important stimulus for the formation of *β*‐sheet peptide‐based nanofibers. Temperature can change the driving force of peptide self‐assembly to regulate the mechanism and process of self‐assembly. Research on PA has shown that the temperature affects the lengths of the formed nanofibers, and the additional thermal energy provided by the annealing process enables the nucleation and rapid growth of nanofibers with a *β*‐sheet secondary structure.^[^ ^64^ ^]^ Moreover, as the temperature increases, the proportion of *β*‐sheet structures formed by self‐assembly increases.^[^ ^65^ ^]^ For example, the amyloid beta peptide C_16_‐KKFFVLK is responsive to temperature changes and unwinds significantly when heated. It self‐assembles into helical ribbons and nanotubes at 20 °C and forms twisted tapes when heated to 55 °C (Figure 5C).^[^ ^66^ ^]^ Since the *β*‐sheet content affects the mechanical rigidity of the hydrogel,^[^ ^67^ ^]^ temperature regulation is crucial when preparing peptide‐based scaffolds with suitable hardness.

#### 4.2.3 Responsiveness to Ions

Metal ions such as Cu^2+^, Ca^2+^, and Zn^2+^ can regulate the functions of proteins. Therefore, ion‐triggered peptide‐based self‐assembling materials have received extensive research attention. Ions can recognize individual polypeptide sequences, promote cross‐linking between molecules, and promote the self‐assembly of polypeptides into stable structures.

The structures and yields of self‐assembling amyloid nanosheets can be fine‐tuned by changing the ionic strength of the aqueous solution.^[^ ^68^ ^]^ Ulijn and co‐workers co‐assembled FFD, a structure‐forming tripeptide with GHK, a functional tripeptide and, through experiments and computations, found that copper ions promoted the assembly of the peptides into hydrogels with highly ordered nanostructures (Figure 5D).^[^ ^69^ ^]^ Clinical trials have shown that treatment with GHK‐Cu can improve skin ulcers in diabetic patients.^[^ ^70^ ^]^ The GHK‐Cu^2+^ complex promotes the wound repair process by modulating the expressions of glycosaminoglycans and proteoglycans related to cell adhesion, migration, and proliferation in the ECM.^[^ ^71^ ^]^ The self‐assembly of peptide‐based nanomaterials also can be induced to show antibacterial activity by the negatively charged lipid membrane.^[^ ^72^ ^]^

#### 4.2.4 Responsiveness to Enzymes

Enzyme biocatalysis has been employed in many studies to induce in situ peptide self‐assembly.^[^ ^73^ ^]^ Precursor molecules are converted into self‐assembling hydrogelators under the action of enzymes and then self‐assemble into fibrous hydrogels.^[^ ^44^ ^]^ An enzymatic hydrogel molecule usually contains a cleavage sequence or an enzyme‐responsive group. The enzyme response often involves enzyme‐catalyzed chemical bond cleavage (e.g., the dephosphorylation of phosphate by phosphatase,^[^ ^74^ ^]^ ester hydrolysis by esterase,^[^ ^75^ ^]^ amide hydrolysis by MMP‐9,^[^ ^76^ ^]^ and subtilisin hydrolysis^[^ ^77^ ^]^). When a pair of enzymes (e.g., kinase/phosphatase^[^ ^78^ ^]^ and thermolysin/subtilisin) is used, both bond‐breaking and bond‐forming reactions are involved in the enzyme‐responsive system. Therefore, the sol‐gel‐sol phase transition can be achieved by an enzymatic switching reaction.

As an example, subtilisin can hydrolyze methyl ester groups to initiate nucleation, promote the early structural growth of self‐assembling peptide derivatives, and finally form longer nanofibers upon the addition of extra enzymes (Figure 5E).^[^ ^77^ ^]^ Moreover, enzymatic self‐assembly enables peptide‐based materials to respond to enzymes that are overexpressed in different diseases, which can improve the treatment.^[^ ^53^ ^]^

### 4.3 Mimicking the Extracellular Matrix

The ECM, which is composed of geometrically arranged collagen nanofibers, acts as a scaffold to guide the cells during tissue remodeling and provides a highly defined microenvironment that is essential for the repair of damaged tissue.^[^ ^79^ ^]^ Self‐assembling peptide‐based hydrogels avoid immunogenicity due to their biodegradability and non‐toxicity; they also spontaneously and rapidly form entangled nanofiber networks without chemical cross‐linking reactions or additional components.^[^ ^80^ ^]^ Therefore, peptide‐based hydrogels prepared by self‐assembly can mimic the structure and function of the native ECM to provide a microenvironment similar to that in vivo.^[^ ^81^ ^]^

For example, sPGP, which is composed of a phosphopeptide and its receptor glycopeptide, self‐assembles into hierarchical nanofibers on the cell membrane surface via an enzymatic reaction. The peptide assemblies act as ECM to rapidly switch a 2D cell sheet to 3D cell spheroids and control cell behavior.^[^ ^82^ ^]^ Peptidyl or protein‐based self‐assembling hydrogels that mimic the natural ECM can promote cell attachment and diffusion^[^ ^83^ ^]^ along with cell proliferation since these hydrogels can anchor cells like the natural ECM.^[^ ^84^ ^]^ The ECM also affects tumor invasion and metastasis. An artificial extracellular matrix (AECM) with the composition BP‐KLVFFK‐GGDGR‐YIGSR was designed to transform into nanofibers entwined to form AECM around solid tumors, thereby preventing further tumor invasion and metastasis.^[^ ^8^ ^]^

Due to their ability to mimic the ECM to regulate cell fate along with their striking resemblance to soft tissues, peptidyl hydrogels are promising biomaterials for wound treatment. Peptidyl hydrogels can target signals within the ECM, respond to ECM stimuli, mimic the ECM, and regulate the tissue microenvironment. These characteristics make peptide‐based self‐assembling materials promising candidates for wound‐healing dressings.

## 5 Rational Design of Peptide‐Based Hydrogels for Wound Treatment

Self‐assembling peptide‐based hydrogels can be easily formulated to carry drugs, cytokines, or cells to the desired location and then broken down into bioactive peptide sequences or natural amino acids that can be used to repair the surrounding tissue. Thus, these hydrogels have been widely employed in various stages of wound healing, including hemostasis, infection and inflammation regulation, cell proliferation, and remodeling (**Table** **3**).

**Table 3:** Examples of self‐assembling peptide‐based materials that have potential in wound healing

Name	Peptide sequence	Features	Potential roles in wound healing stages	Ref.
GHK‐Cu	GHK	Exhibits a high affinity for copper(II) ions	Increases GAG and collagen deposition	[71]
FF8	KRRFFRRK	Targets and self‐assembles on the negatively charged bacterial membranes	Antibacterial	[72]
RADA16‐I	(RADA)_4_	Assembles to form a nanofiber‐based clot when in contact with blood	Rapid hemostasis (≈15 s)	[86, 90, 91]
RATEA16	CH_3_CO‐RATARAEARATARAEA‐CONH_2_	Has a stable *β*‐sheet secondary structure and easily self‐assembles into uniform nanofibers	Rapid hemostasis (≈40 s)	[92]
9‐residue peptide	PSFCFKFEP	Forms “beads‐on‐a‐thread” type nanofibers	Rapid hemostasis (≈20 s)	[87]
I_3_QGK	IIIQGK	Undergoes a sol‐gel transition upon addition of transglutaminase (TGase)	Rapid hemostasis (≈10 s)	[88]
d‐EAK16	Ac‐(AEAEAKAC)_2_‐CONH_2_	Self‐assembles into nanofibers	Rapid hemostasis (≈20 s)	[89]
MAX1	VKVKVKVKV^D^PPTKVKVKVKV‐NH_2_	Has a polycationic, lysine‐rich surface	Has broad‐spectrum antibacterial activity	[97]
MARG1	VKVKVRVKV^D^PPTKVKVRVKV‐NH_2_	Displays positive charge; assembles into a network of *β*‐sheet rich fibrils	Antibacterial	[98]
PEP6R	VKVRVRVRV ^D^PPTRVRVRVKV	Self‐assembling *β*‐hairpin peptides; Displays positive charge	Antibacterial	[99]
D‐W362	^d^W^d^K_3_(QL)_6_^d^K_2_	Contacts with lipid membrane to form stable nanofibers	Antibacterial	[100b]
ASCP	(KIGAKI)_3_‐T^D^PPG‐(KIGAKI)_3_	Self‐assembles into hydrogel under external stimulation	Antibacterial	[100c]
Diphenylalanine	FF	Neutral peptides; Interaction with bacterial membranes leads to membrane permeation and depolarization	Antibacterial	[101]
Fmoc‐AA	Fmoc‐F; FmocFF; FmocFFKK	Ultrashort peptides; Self‐assembles into gels at low concentrations	Antibacterial	[102]
Nap‐AA	Nap‐FFKK; Nap‐FFYp	Ultrashort peptides; Forms supramolecular hydrogels at physiological pH	Antibacterial	[103]
Ag‐Ac‐LK_6_‐NH2	Ag‐Ac‐LIVAGK‐NH_2_	Hydrogel slowly releases silver nanoparticles	Antibacterial	[105]
Se@PEP‐Ru NPs	TGRAKRRMQYNRR	AMP has enhanced stability due to the connection with functionalized selenium nanoparticles	Multi‐component antibacterial; selectively images bacteria	[106]
Dex‐PA	C_16_‐V_2_A_2_E_2_K‐Dex	Nanofiber hydrogel sustainably releases Dex in physiological media.	Localized anti‐inflammatory	[112a]
1‐Dex‐P	Nap‐FFK(Dex)‐Y(H_2_PO_3_)‐OH	Enzymatic self‐assembly; Slowly releases Dex	Anti‐inflammatory	[112b]
IPF‐HYD‐GFFY	IPF‐GFFY	Releases IPF under the action of esterase	Anti‐inflammatory	[113]
VEVE‐Ket	VEVE‐Ket	Slowly releases anti‐inflammatory drugs; shows higher selectivity for cyclooxygenase 2 (COX‐2) than COX‐1	Anti‐inflammatory	[115]
MDPs	K_2_(SL)_6_K_2_	Syringe injectable; Self‐assembles to form nanofibers with structural similarity to native ECM	Angiogenesis	[119]
QK	Ac‐KLTWQELYQLKYKGI‐CONH_2_	Binds to VEGF receptor	Angiogenesis	[120a]
VEGF PA	C_16_‐V_2_A_2_‐K_3_G‐KLTWQELYQLKYKGI‐NH_2_	Self‐assembles into nanofibers; Activates VEGF receptors	Angiogenesis	[120b]
SLanc	KSLSLSLRGSLSLSLKGKLTWQELYQLKYKGI	Self‐assembles into nanofibers; Stimulates VEGF receptors and is easily cleaved by MMP‐2	Angiogenesis	[121c,121d]
PRG, KLT	Ac‐(RADA)_4_GPRGDSGYRGDS‐CONH_2_; Ac‐(RADA)_4_G4KLTWQELYQLKYKGI‐CONH_2_	Functional motifs improve the biological activity of self‐assembling peptides	Angiogenesis	[120e]
NO Gel + GAL	Nap‐FFGGG‐ NO donor	Sustainedly releases NO by addition of *β*‐galactosidase	Angiogenesis	[122]
NapFF‐NO	Nap‐FFGGG‐ NO donor	Releases NO in response to *β*‐galactosidase; Co‐transplants with stem cells	Angiogenesis	[25c]
Ultrashort peptide hydrogels	Ac‐LIVAGK‐NH_2_; Ac‐ILVAGK‐NH_2_	Self‐assembles into spiral fibers as a bionic scaffold	Re‐epithelialization	[130]
RADA_16_‐GRGDS RADA_16_‐YIGSR	AcN‐RADARADARADARADAGRGDS‐CONH_2_; AcN‐RADARADARADARADAYIGSR‐CONH_2_	Self‐assembles into nanofibers under physiological conditions; Enhances cell migration and proliferation	Hemostasis; Re‐epithelialization	[132]
RADA_16_‐IKVAV	RADARADARADARADA‐IKVAV	Self‐assembles into nanofibers; Encapsulates stem cells	Re‐epithelialization	[133]
Angiopoietin‐1‐derived peptide	QHREDGS	Interacts with integrins to promote the proliferation and migration of keratinocytes	Re‐epithelialization; Formation of granulation tissue	[134]
TCP‐25	GKYGFYTHVFRLKKWIQKVIDQFGE	Thrombin‐derived peptide; Can be cleaved into biologically active fragments	Antibacterial; Anti‐inflammatory	[137]
HM‐PA	Lauryl‐VVAGEGD(K‐psb)S‐Am	Mimics the activity of heparin and forms a hydrogel at neutral pH	Angiogenesis; Re‐epithelialization	[138]
BQA‐GGFF	BQA‐GGFF	Mimics neutrophil extracellular traps; Forms a stable hydrogel and emits fluorescence under ROS	Antibacterial; Consumes excessive ROS	[143]
HDMP	bis‐pyrene‐KLVFF‐RLYLRIGRR	Utilizes in situ ligand‐receptor‐induced self‐assembly for recognizing and trapping bacteria	Antibacterial	[145]
FGF@Fiber‐AMP@Peptide	Nap‐GFFKH	Peptides self‐assemble outside the alginatefiber in weak acidic solution (pH ≈ 6.0); Antibiotics can be burst‐released from the peptide hydrogel	Antibacterial; Diminishes inflammation	[146b]

GAG: glycosaminoglycans; AMP: antimicrobial peptide; Dex: dexamethasone; IPF: ibuprofen; Ket: ketoprofen; ECM: extracellular matrix; VEGF: vascular endothelial growth factor; MMP: matrix metalloproteinase; NO: nitric oxide; ROS: reactive oxygen species.

### 5.1 Rapid Hemostasis

**Figure 6:**
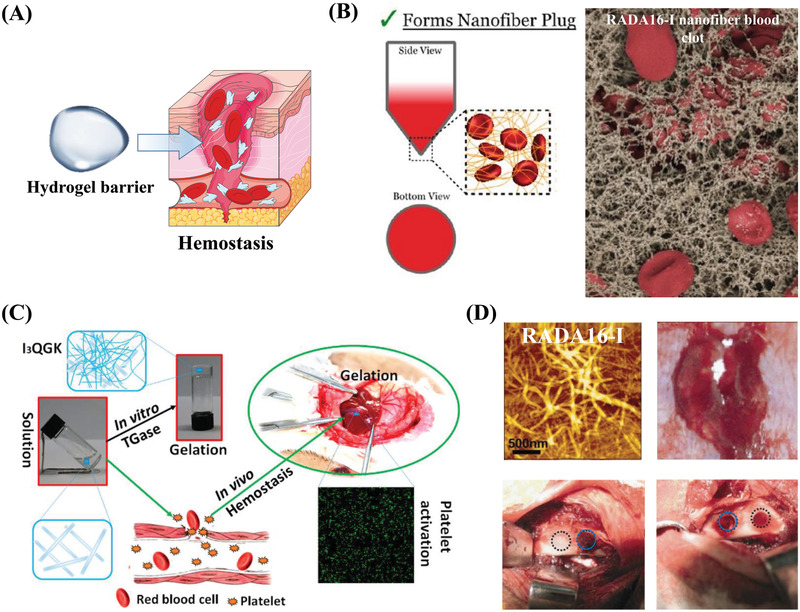
Hemostatic properties of self‐assembling peptides. A) Schematic illustration of self‐assembling peptide barriers for hemostasis. Part of the figure is modified from Servier Medical Art (http://smart.servier.com/), licensed under a Creative Common Attribution 3.0 Generic License. B) Hemostatic mechanism of a peptide nanofibrous hydrogel (RADA16‐I). Suspension assay shows that the nanofibers entangle with red blood cells to keep them in suspension, and the scanning electron microscopy (SEM) image shows that interwoven nanofibers entrap blood components and form clots to speed up hemostasis. Reproduced with permission.^[^ ^86^ ^]^ Copyright 2015, American Chemical Society. C) Hemostatic properties and mechanism of a short‐peptide (I_3_QGK) hydrogel. I_3_QGK assembles into rigid hydrogels in the presence of transglutaminase (TGase) and shows adequate hemostasis by gelling blood and promoting platelet adhesion in a liver trauma model. Reproduced with permission.^[^ ^88^ ^]^ Copyright 2016, American Chemical Society. D) Hemostatic properties of a longer peptide (RADA16‐I). AFM images show that the blood of the rabbit's middle auricular artery can induce the formation of a blood‐hydrogel. Hemostasis was achieved in 10 s (blue circles) in a cancellous ilium bone defect model. Reproduced with permission.^[^ ^90^ ^]^ Copyright 2015, American Chemical Society.

As a hemostatic agent, hydrogels can stop bleeding through both physical and chemical processes, resulting in excellent performance and therapeutic potential for bleeding control.^[^ ^85^ ^]^ Peptide‐based nanofibers can adhere to the wound site and self‐assemble into a hydrogel barrier to seal the wound and achieve complete hemostasis (**Figure** **6**A). During hemostasis, peptide‐based nanofibers may form clots that entrap the blood components and promote platelet adhesion once they encounter blood (Figure 6B).^[^ ^86^ ^]^ The physical entrapment of blood components in the nanofiber network is similar to the behavior of a naturally coagulated fibrin clot.^[^ ^86^ ^]^ Therefore, peptide‐based hydrogels have been reported to stop bleeding in a significantly shorter time (within 1 min) compared with other hemostatic materials (e.g., gauze and chitosan).^[^ ^87^ ^]^

A short self‐assembling peptide (I_3_QGK) was reported to form a hydrogel under the regulation of transglutaminase (TGase) and showed rapid and effective hemostasis in a liver trauma model along with low cytotoxicity and no nonspecific immunological responses (Figure 6C).^[^ ^88^ ^]^ The longer ionic complementary self‐assembling peptides EAK16 and (RADA)_4_ are also used for hemostasis and show rapid hemostasis in transverse liver experiments.^[^ ^89^ ^]^ Interestingly, the time required for _D_‐EAK16 to form a hydrogel under physiological conditions (≈20 s) is significantly shorter than that in water (16 h).^[^ ^89^ ^]^ (RADA)_4_ exhibited immediate hemostasis (≈10 s) in a cancellous ilium bone defect (Figure 6D),^[^ ^90^ ^]^ and (RADA)_4_ nanofibers showed good hemocompatibility with no significant red blood cell or platelet lesions observed after use.^[^ ^91^ ^]^ Optimizing the acidic pH of RADA by replacing the strongly acidic aspartic acid residues with weakly acidic glutamic acid and basic threonine residues effectively shortened hemostasis time in the rabbit liver injury wound model.^[^ ^92^ ^]^ Polypeptide modification can be employed to obtain hydrogels with robust hemostatic properties. For example, supercharged polypeptides (SUPs) and biomimetic synthetic surfactants can form non‐swelling adhesive materials through electrostatic complexation.^[^ ^93^ ^]^ SUP glue exhibited tissue adhesion and hemostatic effects in pig liver and kidney bleeding models, with bleeding inhibited within 10 s.^[^ ^93^ ^]^

Peptide‐based materials have excellent potential for wound hemostasis due to their ability to self‐assemble into nanofibrous hydrogels. However, most hemostatic materials are only suitable for wounds with regular shapes and low blood flow. Bleeding control is difficult in irregularly shaped wounds (e.g., wounds located in moving parts of the body) because of the high blood flow and difficulty in effectively applying treatment materials to the wound site.^[^ ^94^ ^]^ Therefore, it is important to design suitable peptide‐based hemostatic hydrogels for specific applications in the future. Future studies should focus on functional peptide‐based materials with high viscosity and stretchability that can self‐assemble in situ to fit bleeding tissues.

### 5.2 Preventing Infection in Wounds

**Figure 7:**
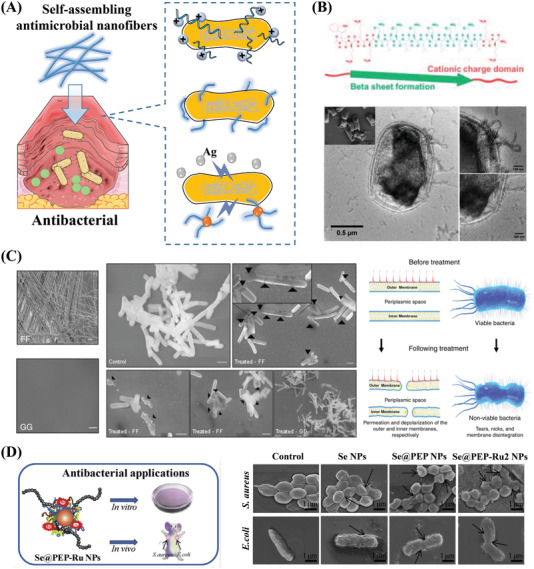
Antibacterial properties of self‐assembling peptides. A) Schematic illustration of three types of antibacterial peptide‐based self‐assembling materials: Materials that kill bacteria through cationic properties, assembly structures, and fungicide delivery. Part of the figure is modified from Servier Medical Art (http://smart.servier.com/), licensed under a Creative Common Attribution 3.0 Generic License. B) A self‐assembling cationic peptide (D‐W362: ^d^W^d^K_3_(QL)_6_^d^K_2_) destroys bacterial cell membranes. SEM and negatively stained TEM images show that D‐W362 targets the bacterial cell membrane and leads to local membrane deformation and rupture. Reproduced with permission.^[^ ^100b^ ^]^ Copyright 2018, American Chemical Society. C) The self‐assembling neutral peptide FF deforms and ruptures cell membranes. SEM images show that FF self‐assembles into nanofibers. High‐resolution SEM shows that the cell membranes of bacteria treated with FF were damaged compared to those treated by GG, which does not self‐assemble. A schematic of the proposed mechanism shows that FF leads to severe changes in membrane morphology. Reproduced with permission.^[^ ^101^ ^]^ Copyright 2017, Springer Nature. D) Antimicrobial peptides linked to functionalized nanoparticles (Se@PEP‐Ru NPs) for the targeted therapy of bacterial infections. SEM images reveal that the Se@PEP‐Ru2 NPs cause more morphological changes in both Gram‐positive and Gram‐negative bacterial cell membranes compared to the control groups. The NPs kill bacteria by severely disrupting the integrity of the bacterial cell and cytoplasmic membranes. Reproduced with permission.^[^ ^106^ ^]^ Copyright 2017, Elsevier Ltd.

The most common challenge in wound healing is preventing infection caused by the invasion of microorganisms, especially in diabetic wounds. Antibiotic‐resistant strains such as *Staphylococcus aureus* and *Pseudomonas aeruginosa* can cause postoperative infections, particularly in burn and chronic wounds, resulting in delayed wound healing and increased mortality.^[^ ^95^ ^]^ To address this issue, peptide‐based materials have been developed as effective defensive weapons against infection. For example, bacteria show no resistance or lower resistance to antimicrobial peptides (AMPs) compared to antibiotics, which is beneficial for aseptic wound healing.^[^ ^96^ ^]^ Current treatment approaches typically take advantage of the cationic properties, self‐assembling structures, and fungicide‐carrying capabilities of different peptide‐based material systems to prevent infection and treat infected wounds (**Figure** **7**A).

#### 5.2.1 Cation‐Rich Antimicrobial Peptides

Cation‐rich self‐assembling peptides that are rich in lysine and arginine have polycationic surfaces that exhibit antibacterial activity against a broad spectrum of Gram‐positive and Gram‐negative bacteria. Cation‐rich AMPs combine with bacterial cell membranes to destroy the membrane potential, alter membrane permeability, and leakage of metabolites, ultimately leading to bacterial cell death.

For example, the cationic self‐assembling hydrogels MAX1^[^ ^97^ ^]^ and MARG1^[^ ^98^ ^]^ have inherent antibacterial activity. The peptide fibers of MAX1 are composed entirely of lysine residues, providing resistance to bacterial infection. In MARG1, the addition of arginine results in enhanced antibacterial properties.^[^ ^98^ ^]^ The content of arginine affects the performance of the antibacterial peptide hydrogel. Four peptides with different arginine contents showed different mechanical, antibacterial, and hemolytic properties.^[^ ^99^ ^]^ Many other self‐assembling peptides rich in lysine and arginine (e.g., multi‐domain peptides and lipopeptides) also exhibit broad‐spectrum antibacterial activity by destroying bacterial cell membranes.^[^ ^100^ ^]^ For example, D‐W362, a cationic multidomain peptide (MDP), deforms and ruptures the bacterial cell membrane through interactions between the D‐W362 nanofibers and the membrane (Figure 7B).^[^ ^100b^ ^]^ Unlike traditional AMPs, self‐assembling cationic AMPs have good hemocompatibility, are membrane selective, and can harmlessly cross mammalian cell membranes; thus, they will not have toxic effects in normal cells.^[^ ^100b^ ^]^

#### 5.2.2 Antimicrobial Self‐Assembling Peptides

Uncharged peptides can also self‐assemble into nanofibers that exhibit antibacterial activity through the destruction of bacterial cell membranes. The simplest neutral self‐assembling antibacterial peptide is FF.^[^ ^101^ ^]^ FF treatment leads to the permeation and depolarization of the outer and inner membranes of bacterial cells, as indicated by the observation of numerous nicks and tears in the membrane (Figure 7C).^[^ ^101^ ^]^ It is worth noting that it is the self‐assembly characteristics of FF itself that endow it with antibacterial activity. In contrast, GG, which does not self‐assemble, shows no antibacterial activity (Figure 7C).^[^ ^101^ ^]^ Therefore, chemical modifications based on FF can improve the potency of short self‐assembling AMP agents.

Modifying FF with Fmoc, Nap, and other amino acids can produce antibacterial effects by forming fibrous structures that destroy the bacterial cell membrane. Several Fmoc‐based peptides (e.g., FmocF and FmocFF) have shown activity against most antibiotic‐resistant biofilm phenotypes. The addition of cationic peptides to these Fmoc‐based agents (e.g., FmocFFKK, FmocFFFKK, and FmocFFOO) allows the short peptides to exert antibacterial effects at lower concentrations.^[^ ^102^ ^]^ Substituting naphthalene (Nap) for Fmoc in the above materials retains the bactericidal properties while mitigating the potentially toxic effects of Fmoc.^[^ ^103^ ^]^ Amino acid‐modified FF materials also exhibit antibacterial properties. For example, the AMP KRRFFRRK (FF8) can self‐assemble into nanofibers on the negatively charged lipid membranes and destroy the cell membrane of Gram‐negative bacteria to achieve an antibacterial effect.^[^ ^72^ ^]^

#### 5.2.3 Combination of Peptides with Fungicides

The combination of peptide‐based materials with fungicides has become a promising approach for treating chronic wounds and injuries. Metal‐based NPs are common antibacterial agents. One representative example is silver (Ag) NPs.^[^ ^104^ ^]^ Silver compounds have broad antibacterial activity against up to 12 Gram‐positive and Gram‐negative bacteria along with some advantages over traditional antibiotics. For self‐assembling lipopeptides that do not have antibacterial activity, Ag doping can create an antibacterial hydrogel. Take Ag‐Ac‐LIVAGK‐NH_2_ (Ag‐Ac‐LK_6_‐NH_2_) as an example; the mechanical stiffness of the hydrogel increases in the presence of Ag NPs.^[^ ^105^ ^]^ The sustained release of Ag NPs in situ prevents bacterial infection, especially against *P. aeruginosa*.^[^ ^105^ ^]^

Antibacterial peptides have also been combined with other metal‐based NPs to enhance the antibacterial activity. For example, the antibacterial peptide UBI29‐41 (PEP) is associated with functionalized selenium NPs and combines an Ru complex.^[^ ^106^ ^]^ NPs destroy the bacterial membrane to kill the bacteria, and PEP can target bacteria to improve the bactericidal effects of NPs.^[^ ^106^ ^]^ Based on in vivo antibacterial experiments, PEP shows effective antibacterial action and promotes the healing of infected wounds (Figure 7D).^[^ ^106^ ^]^ Other functional fungicides such as gold NPs,^[^ ^107^ ^]^ polyphenols,^[^ ^108^ ^]^ and fluoride^[^ ^109^ ^]^ also show excellent antibacterial effects. Combining these fungicides with self‐assembling peptide hydrogels is expected to further improve the antibacterial performance. These in situ fungicide‐releasing peptide‐based hydrogels can prevent wound infection (e.g., in burn wounds) and reduce sustained inflammation in chronic wounds.

### 5.3 Anti‐Inflammatory Activity

Once a severe bacterial infection occurs, the inflammatory response may escalate to a detrimental level, resulting in long‐term, systemic inflammation as the wound progresses. In this case, effective strategies are needed to fight inflammation. Anti‐inflammatory drugs (both steroids and non‐steroids) are commonly used to treat inflammation in chronic wounds. However, oral drug administration can cause adverse reactions in the gastrointestinal tract, kidneys, and cardiovascular systems, while local nanocarrier administration results in short retention time and the uncontrollable release of anti‐inflammatory drugs in wounds.^[^ ^110^ ^]^ Topical formulations of peptide‐based self‐assembling hydrogels can encapsulate traditional anti‐inflammatory drugs in peptide assemblies to achieve controlled drug release and improve the biological transmission and bioavailability of anti‐inflammatory drugs, thereby reducing implant‐related inflammation and decreasing toxic side effects during the anti‐inflammatory process.

#### 5.3.1 Co‐Assembly of Peptides and Anti‐Inflammatory Drugs

Co‐assembly is an effective strategy to enhance the therapeutic effects of anti‐inflammatory drugs while also reducing their adverse side effects. The steroid dexamethasone (Dex) is an effective anti‐inflammatory drug and an immune response inhibitor. Liang and co‐workers developed a strategy for the intracellular co‐assembly of peptides and Dex to enhance the anti‐inflammation capacity of Dex.^[^ ^111^ ^]^ The overexpressed ALP on the membranes of inflammatory macrophages served as a catalyst to dephosphorylate the peptide precursor (Nap‐Phe‐Phe‐Tyr(H_2_PO_3_)‐OH) and Dex sodium phosphate, which were then co‐assembled inside the cells. In experiments, the ALP‐induced intracellular co‐assembly greatly enhanced the anti‐inflammation efficacy of Dex in inflammatory cells.^[^ ^111^ ^]^ This method provides new insights for improving the efficacy of anti‐inflammatory drugs.

#### 5.3.2 Chemical Modification of Peptides with Anti‐Inflammatory Drugs

Chemically modified bio‐conjugation strategies can be applied to anti‐inflammatory drugs to endow them with additional beneficial properties. Self‐assembling peptides and drugs linked by chemical bonds have good assembly efficiency, stability, and anti‐inflammatory effects. The peptide Nap‐Phe‐Phe‐Lys‐Tyr(H_2_PO_3_)‐OH and PAs can be covalently conjugated to Dex through an ester bond and a hydrolyzable hydrazone linkage, respectively.^[^ ^112^ ^]^ In addition, ibuprofen can be linked with the peptide GFFY through a cleavable ester bond and spontaneously form a hydrogel.^[^ ^113^ ^]^ Due to the enhanced tissue retention and enzyme activity at the inflammation site, hydrogels can slowly release anti‐inflammatory drugs and facilitate localized anti‐inflammatory activity. Furthermore, various nonsteroidal anti‐inflammatory drugs (NSAIDs) can conjugate with self‐assembling short peptide sequences (sequences containing Phe‐Phe).^[^ ^114^ ^]^ The covalent coupling of self‐assembling short peptides and NSAIDs has been shown to improve selectivity for inhibiting cyclooxygenase‐2.^[^ ^114^, ^115^ ^]^ These molecules can assemble into hydrogels and effectively retain the activity of NSAIDs, providing a new type of hydrogel for anti‐inflammatory treatment. It is worth noting that an unwanted side effect of Dex treatment is that the reduction of VEGF in the surrounding tissues hinders angiogenesis and delays wound healing.^[^ ^116^ ^]^ Therefore, NSAIDs may have more potential for the treatment of wound inflammation.

Overall, peptide‐based hydrogels obtained by co‐assembly or coupling with anti‐inflammatory drugs can be used to treat local inflammation while enhancing retention time and reducing side effects without affecting drug activity. More importantly, since wound healing is mediated by various immune cells and signaling molecules,^[^ ^117^ ^]^ the inflammation‐related immune response has become an important topic of study.^[^ ^24b^ ^]^

### 5.4 Proliferation and Remodeling

After wound infection is controlled, the subsequent skin and tissue proliferation stage is important for successful wound healing. This stage involves angiogenesis, fibroblast and keratinocyte proliferation, and collagen deposition. Peptide‐based hydrogels with properties similar to the ECM have been widely developed as 3D scaffolds for tissue engineering.^[^ ^81^ ^]^

#### 5.4.1 Peptide and Peptide‐Nitric Oxide Hydrogels for Angiogenesis

**Figure 8:**
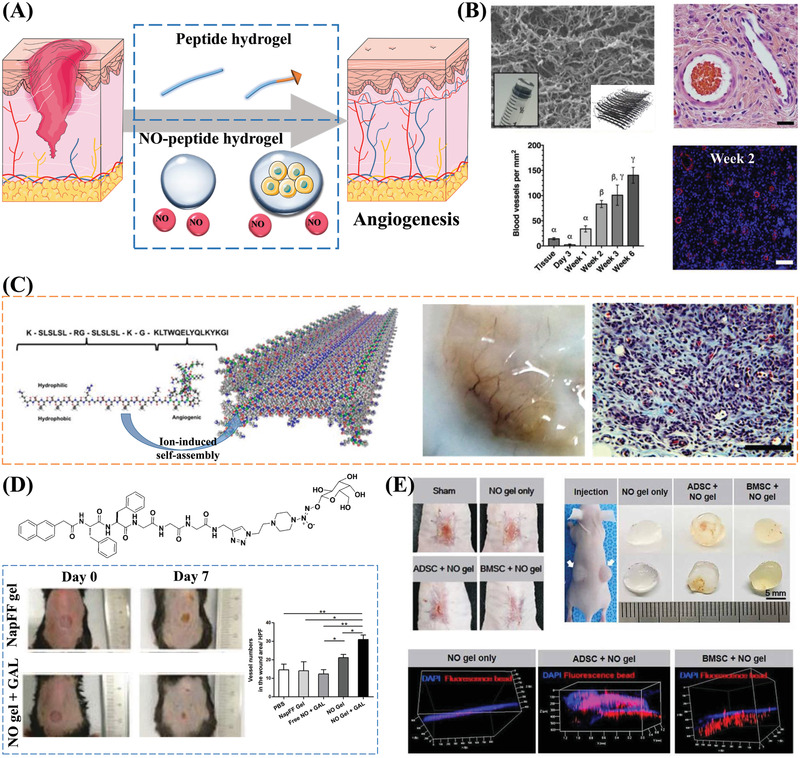
Angiogenic properties of self‐assembling peptides. A) Schematic illustration of self‐assembling peptide and NO‐peptide hydrogels for promoting angiogenesis. Part of the figure is modified from Servier Medical Art (http://smart.servier.com/), licensed under a Creative Common Attribution 3.0 Generic License. B) The site of the subcutaneous injection of the peptide hydrogel K_2_(SL)_6_K_2_ is highly vascularized. The SEM image shows the fiber network structure formed by the self‐assembly of peptides. The H&E staining image of blood vessels in the hydrogel and plot of the number of blood vessels show that the implantation of the peptide hydrogel can promote angiogenesis. Reproduced with permission.^[^ ^119^ ^]^ Copyright 2018, Elsevier Ltd. C) A VEGF‐binding self‐assembling peptide activates receptors to promote angiogenesis. After the subcutaneous implantation of the peptide hydrogel scaffolds, a large number of blood vessels can be seen. The Masson's Trichrome staining image further indicates the formation of blood vessels. Reproduced with permission.^[^ ^120c^ ^]^ Copyright 2015, American Chemical Society. D) NO‐releasing hydrogel promotes wound angiogenesis: *β*‐galactosidase triggers NO release and promotes peptide gelation; Number of stained micro‐vessels per HPF shows that NO gel + GAL group significantly promotes angiogenesis in wounded skin. Reproduced with permission.^[^ ^122^ ^]^ Copyright 2013, Royal Society of Chemistry. E) NO‐gel and MSCs synergistically induce neovascularization. Images from in vivo wound healing experiments show that wound closure occurs faster in the BMSC‐embedded NO‐gel group compared to the other experimental groups. The gel plug angiogenesis assay results and 3D visualization of perfusable vessel formation indicate the ability of the NO‐gel to induce angiogenesis. Reproduced with permission.^[^ ^127^ ^]^ Copyright 2020, AAAS.

Neovascularization can significantly improve the wound microenvironment and promote the process of wound closure. Without sufficient blood supply to directly exchange oxygen, nutrients, and waste, new tissue will not grow or repair the wound.^[^ ^118^ ^]^ Self‐assembling nanofibrous matrices show excellent potential for application in vascular problems. Interestingly, self‐assembling peptides can promote the angiogenesis process in wounds based on their biological activity and ability to deliver special chemical signaling molecules (**Figure** **8**A).

Peptide‐based nanofibrous hydrogel networks can promote angiogenesis without the need for additives. 1 week after the subcutaneous injection of a MDP hydrogel, the hydrogel site was highly vascularized (Figure 8B).^[^ ^119^ ^]^ The pro‐angiogenesis effect is related to the acute inflammatory response after hydrogel injection, which causes the hydrogel‐recruited cells to secrete cytokines and growth factors, which are transferred to the tissues where they start cascade reactions.^[^ ^119^ ^]^ VEGF facilitates the angiogenesis process in wounds, and many studies have regulated the angiogenic response by targeting VEGF and its receptors.^[^ ^120^ ^]^ Compared to other peptide‐based hydrogels, VEGF‐based peptide hydrogels have better angiogenesis‐promoting properties. The peptide QK, which mimics the fragment on VEGF, can bind to the VEGF receptor and is a potential angiogenesis agonist.^[^ ^120a^ ^]^ Moreover, peptide‐based hydrogels with MMP‐sensitive sites can control the release of VEGF to promote angiogenesis. Slanc, a complex peptide composed of QK and MDP with an MMP‐2 cleavage sequence,^[^ ^120c^ ^]^ forms a hydrogel upon injection, and the hydrogel releases VEGF mimetics after MMP‐2 digestion. These VEGF mimetics further bind to VEGF receptors to promote angiogenesis (Figure 8C).^[^ ^120c^ ^]^ Implants can be degraded and then replaced by native tissues after exerting their desired effects without causing additional inflammation. It also has an excellent angiogenesis‐promoting effect in severe ischemic tissues.^[^ ^120c,121d^ ^]^ Conjugating QK with self‐assembling peptides can effectively promote angiogenesis. For example, QK was combined with peptide amphiphile (PA)^[^ ^120b^ ^]^ and RADA16, an aliphatic self‐assembling peptide,^[^ ^120e^ ^]^ to promote angiogenesis in ischemic tissue.

Nitric oxide (NO) is an essential signaling molecule that promotes angiogenesis. NO is usually an endogenous vasodilator produced by intact endothelial cells of the blood vessel intima. NO is a novel molecule of interest in wound healing because it has been shown to facilitate wound repair by promoting EC growth and migration and promoting new blood vessel generation from existing vessels.^[^ ^121^ ^]^ Therefore, combining short peptide sequences with NO donors is a useful strategy for constructing self‐assembling hydrogels. For example, an enzyme‐controllable NO‐releasing hydrogelator was formed from a sugar‐caged NO donor and a peptide‐containing sequence (Nap‐FFGGG).^[^ ^25c^ ^]^ The hydrogelator, which was generated by the Cu (I)‐catalyzed click reaction between an alkyne (containing a short peptide derivative) and an azide (containing a caged NO donor), can release NO in the presence of *β*‐galactosidase.^[^ ^122^ ^]^ The local release of NO in the wounded skin significantly promotes neo‐vascularization, thereby accelerating the wound healing process (Figure 8D).^[^ ^122^ ^]^

The use of NO‐gels carrying stem cells is another strategy for promoting angiogenesis. The presence of NO promotes angiogenesis by regulating paracrine effects,^[^ ^123^ ^]^ endothelial differentiation, and pericyte function^[^ ^124^ ^]^ in human mesenchymal stem cells (hMSCs).^[^ ^125^ ^]^ The co‐transplantation of gelatin‐based hydrogels and MSCs had a marked pro‐angiogenic effect.^[^ ^126^ ^]^ The release of NO and hMSCs synergistically induced the formation of new blood vessels and promoted wound healing (Figure 8E).^[^ ^127^ ^]^ Hydrogels carrying different types of stem cells, including adipose‐derived MSCs (ADSCs) and bone marrow MSCs (BMSCs), may result in different healing speeds (e.g., wound closure occurs faster in BMSC‐embedded NO gels than in ADSC‐embedded NO gels), revealing the importance of appropriate stem cell selection in co‐culture systems for promoting angiogenesis.

#### 5.4.2 Peptide‐Based Fiber Network Scaffolds for Re‐Epithelialization

**Figure 9:**
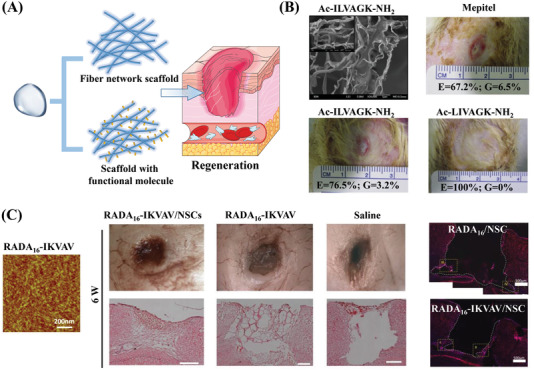
Re‐epithelialization properties of fibrous scaffolds of self‐assembling peptides. A) Schematic diagram of self‐assembling peptide scaffolds and self‐assembling peptides binding functional molecules for promoting regeneration. Part of the figure is modified from Servier Medical Art (http://smart.servier.com/), licensed under a Creative Common Attribution 3.0 Generic License. B) A self‐assembling short‐peptide scaffold promotes the regeneration of burned epidermal tissue. The field‐emission SEM image shows the ECM‐like nanofibers and sheet‐like structures of the short peptide Ac‐ILVAGK‐NH_2_. Images showing the measurement of wound re‐epithelialization (E) and granulation (G) demonstrate that the hydrogels accelerate the regeneration of new epidermal tissue. Reproduced with permission.^[^ ^130^ ^]^ Copyright 2014, Elsevier Ltd. C) A peptide‐based fiber scaffold (RADA_16_) combined with laminin‐derived IKVAV promotes brain tissue regeneration. The AFM image shows the nanofibrous structure of the peptide. Morphological examination and H/E staining images of brain wound defects show that the size of the wound decreases after RADA_16_‐IKVAV treatment, and newly formed ECM tissue can be seen. The immunohistochemistry results also demonstrate the regeneration of neural brain tissue. Reproduced with permission.^[^ ^133^ ^]^ Copyright 2012, Elsevier Ltd.

Re‐epithelialization during wound healing can promote wound closure and reduce the risk of persistent inflammation. The inability to re‐epithelialize is a characteristic of chronic non‐healing wounds and prevents wounds from healing in an orderly and timely manner.^[^ ^128^ ^]^ Due to their nanofibrous geometries, peptide‐based self‐assembling hydrogels provide stable fibrous scaffolds with characteristics similar to the natural ECM, potentially mimicking the 3D ECM microenvironment in vivo. Therefore, self‐assembling peptides exhibit distinctive advantages, including the ability to quickly integrate with host tissues and promote cell proliferation and migration.^[^ ^129^ ^]^ Peptide‐based hydrogel scaffolds promote cell proliferation and migration both with and without biologically active sequences, thus contributing to the re‐epithelialization of various wounds (**Figure** **9**A).

Hydrogels formed via the self‐assembly of short peptides can provide a stable nanofibrous network structure while promoting epithelial and dermal regeneration.^[^ ^130^ ^]^ Compared with other dressings on the market, two ultra‐short aliphatic peptide hydrogels (Ac‐ILVAGK‐NH_2_ and Ac‐LIVAGK‐NH_2_) with similar structures accelerated the autolytic debridement of the necrotic eschar tissue and the epithelial regeneration of burn wounds without exacerbating the inflammatory response (Figure 9B).^[^ ^130^ ^]^ By optimizing the peptide sequence through the addition of cysteine (LIVAGKC), the retention time of the hydrogel was improved via disulfide bond crosslinking. When applied as a novel dressing, this LIVAGKC hydrogel achieved the complete re‐epithelialization of full‐thickness excision wounds in mice.^[^ ^131^ ^]^

Although self‐assembling peptides show promise as scaffolds, their specific cell interactions are limited. The introduction of bioactive epitopes into peptide‐based hydrogels can promote cell adhesion and proliferation. Due to their dual roles as structural and adhesive frameworks, RGDS, YIGSR, and IKVAV have been studied as ECM mimetics. They can be combined with self‐assembling peptide sequences to improve cell adhesion, thereby providing a more effective scaffold for re‐epithelialization. For example, combining RADA_16_ with RGDS and YIGSR further promoted liver tissue regeneration compared to RADA_16_ alone.^[^ ^132^ ^]^ In addition, a RADA_16_‐IKVAV hydrogel encapsulating neural stem cells significantly promoted brain neural tissue repair and regeneration (Figure 9C).^[^ ^133^ ^]^

Chronic wounds, such as wounds in diabetic patients, usually show high levels of reactive oxygen species (ROS) and enhanced ECM degradation resulting from the elevated level of MMPs, which leads to impaired angiogenesis and cell migration.^[^ ^134^ ^]^ Recently, the peptide QHREDGS was found to accelerate wound closure in diabetic mice by enhancing re‐epithelialization and granulation tissue formation.^[^ ^134^ ^]^ However, further analysis is required to address the problem of uncontrolled angiogenesis and impaired re‐epithelialization in chronic wounds.

### 5.5 Advanced Self‐Assembling Peptide‐Based Materials for Spatiotemporally Controlled Wound Healing

**Figure 10:**
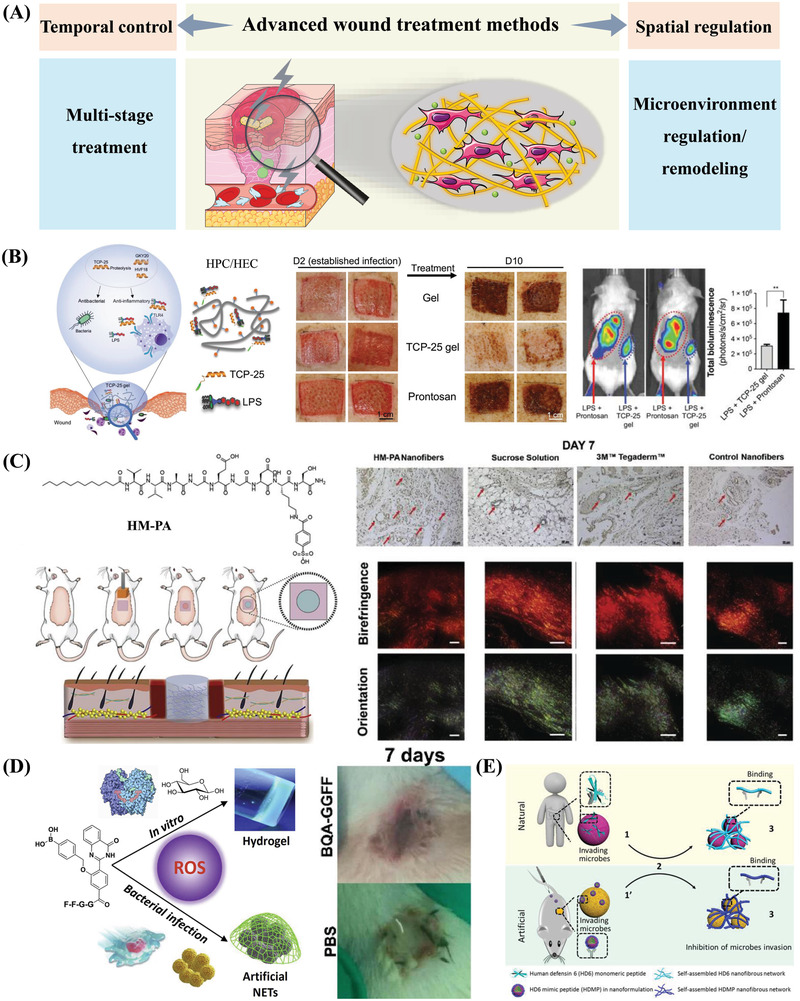
Peptide‐based self‐assembling materials for spatiotemporally controlled wound healing. A) Schematic diagram of wound treatment with spatial and temporal control. Part of the figure is modified from Servier Medical Art (http://smart.servier.com/), licensed under a Creative Common Attribution 3.0 Generic License. Multiple functions of time‐controllable peptide‐based materials for wound treatment. B) TCP‐25 hydrogel acts on wound infection and the accompanying inflammation. Photographic images of the treatment of bacterially infected mini‐pig wounds show that TCP‐25 hydrogel leads to the faster healing of infected wounds compared to common wound treatment agents. In vivo inflammation imaging also shows that TCP‐25 hydrogel has significant anti‐inflammatory activity. Reproduced with permission.^[^ ^137^ ^]^ Copyright 2020, AAAS. C) HM‐PA hydrogel facilitates wound angiogenesis and later collagen regeneration. Staining images of blood vessels show that peptide nanofiber treatment greatly enhances angiogenesis at the wound site at day 7. Picrosirius red staining images show that peptide nanofiber scaffolds have a good wound collagen deposition ability. Reproduced with permission.^[^ ^138^ ^]^ Copyright 2017, Elsevier Ltd. Spatially controlled regulation of the immune microenvironment by peptide‐based materials in wound therapy. D) BQA‐GGFF mimics the immune process of neutrophil extracellular traps (NETs) to regulate the wound microenvironment and promote wound healing. BQA‐GGFF self‐assembles into fluorescent nanofibers triggered by inflammatory ROS. Experiments using a catheter‐related infection model show that the wounds healed faster in the group treated with BQA‐GGFF compared to the PBS control. Reproduced with permission.^[^ ^143^ ^]^ Copyright 2020, Elsevier Ltd. E) Self‐assembling cationic antimicrobial peptides mimic human defensin 6 (HD_6_) to trap bacteria. Schematic diagram of HDMP imitating HD_6_ in the human body to capture bacteria. HDMP NPs assemble into nanofibers in situ on the surface of *S. aureus* and trap them to inhibit bacterial invasion. Reproduced with permission.^[^ ^145^ ^]^ Copyright 2020, AAAS.

In clinical applications, the tissue environment of wounds is delicate and complicated, and wound dressings that have only a single role in wound healing are usually insufficient. Thus, the design of more advanced peptide‐based materials that provide spatiotemporal control over multiple stages of the wound healing process is critical. Peptide‐based hydrogels can provide effective temporal control for long‐term wound treatment, sustained drug release, and controlled degradation. Meanwhile, peptide‐based hydrogels can provide spatial control as follows: The soft hydrogel can fit the edges of tissue; the nanofibrous structure mimics the natural ECM; and the hydrogel serves as a scaffold to regulate the wound microenvironment. Therefore, taking advantage of their stimuli‐responsive self‐assembly characteristics, the personalized design of peptide‐based hydrogels shows considerable potential for multimodal treatments that respond to the dynamic changes within wound tissues. Furthermore, stable fibrous scaffolds of well‐designed hydrogel dressings also regulate and remodel the wound microenvironment. This section focuses on two approaches for using peptide‐based materials for advanced wound therapy with spatiotemporal control: Multimodal wound therapy and wound microenvironment regulation (**Figure** **10**A).

#### 5.5.1 Multimodal Peptide‐Based Hydrogels for Wound Healing

It shows a long‐term and continuous process as wound healing progresses, involving multiple stages of hemostasis, infection and inflammation response, angiogenesis, and re‐epithelialization. Although we mainly focus on designing peptide‐based materials for a specific stage of wound healing, the peptide‐based hydrogels mentioned above may affect the entire wound healing process over a relatively long period of time. By rationally designing molecules, the hydrogels can provide time‐controllable, multimodal treatments during different stages of wound healing. In recent years, gel dressings have been developed with long‐term, multiple therapeutic effects in wound treatment.^[^ ^15^, ^25^, ^135^ ^]^

Increasing evidence suggests that many AMPs have multiple effects in wounds. In addition to antibacterial effects, they also have various immunomodulatory roles and promote angiogenesis.^[^ ^136^ ^]^ In addition, AMPs activate the host defense peptides (HDPs), which are members of the innate immune system. A series of thrombin‐derived peptides (GKY25, HVF18, and TCP‐25) that imitate HDPs was found to promote the healing of various wounds by preventing infection and the accompanying inflammation. In one study, TCP‐25‐functionalized hydrogels that imitate endogenous HDP in wounds were found to have two therapeutic effects: They combined with bacterial LPS to kill bacteria and prevented LPS‐induced inflammation (Figure 10B).^[^ ^137^ ^]^ In another study, a peptide‐based hydrogel scaffold (HM‐PA) that mimics heparin facilitated wound healing by improving a key shortcoming of heparin (its rapid degradation in biological environments).^[^ ^138^ ^]^ The HM‐PA hydrogel increased angiogenic activity during early regeneration and promoted re‐epithelialization and granulation tissue formation in late‐stage burns (Figure 10C).^[^ ^138^ ^]^

Notably, the healing processes of acute and chronically infected wounds are different. The long‐term inflammation associated with chronic wounds inhibits the formation of blood vessels and hinders the regeneration of wound tissues, causing it to take longer for wounds to heal.^[^ ^25d^ ^]^ Therefore, further study on the design of time‐controllable peptide‐based hydrogels to control inflammation in chronic wounds and regulate wound reconstruction is urgently needed.

#### 5.5.2 Peptide‐Based Hydrogels for Regulating and Remodeling the Wound Microenvironment

The dynamic wound healing process involves surrounding blood vessels, immune cells, biomolecules, and the ECM, which create a specific wound microenvironment.^[^ ^139^ ^]^ The complexity of the wound healing mechanism highlights the importance of targeting the wound microenvironment, which represents a new and promising direction to promote wound healing.^[^ ^139^, ^140^ ^]^

Many peptides can self‐assemble to form entangled nanofiber networks in hydrogels, which spatially mimic the ECM and regulate the wound microenvironment to achieve wound treatment.^[^ ^80a^ ^]^ In recent years, many peptide hydrogels that mimic the ECM have been commercialized. For instance, the nanofibrous peptide hydrogel PuraStat was clinically proven to be safe and effective in controlling different types of gastrointestinal hemorrhage.^[^ ^141^ ^]^ RADA16, a nanofibrous scaffolding material that imitates natural protein motifs used for tissue engineering and regenerative applications, has been commercialized as PuraMatrix.^[^ ^142^ ^]^ Moreover, reasonably designed self‐assembling peptide‐based materials can mimic the natural immune microenvironment to regulate infected wounds. Recently, Gao and co‐workers^[^ ^143^ ^]^ designed BQA‐GGFF, a peptide‐based quinazolinone derivative that imitates the innate immune process of neutrophil extracellular traps (NETs). At the site of bacterial infection in vivo, ROS produced by inflammation successfully induced the peptide to self‐assemble into nanofibers in situ to trap bacteria, prevent infection, and reduce inflammation, promoting faster healing in infected wounds (Figure 10D).^[^ ^143^ ^]^ This study inspired the application of self‐assembling peptides to mimic the wound immune tissue microenvironment and achieve wound treatment with spatiotemporal control. In humans, *α*‐defensins can recognize invading microbes and effectively inhibit infection through ordered self‐assembly into nanofiber networks that surround and wrap bacteria.^[^ ^144^ ^]^ Inspired by the AMP human defensin‐6 (HD6), Wang and co‐workers designed HDMP, a biomimetic peptide containing a ligand peptide sequence (RLYLRIGRR) that can bind to Gram‐positive bacteria and a self‐assembly sequence (KLVFF).^[^ ^145^ ^]^ HDMP specifically recognizes *S. aureus* through ligand‐receptor interactions and assembles in situ into nanofibers on the surface of bacteria to capture and wrap the bacteria, preventing the bacteria from invading host cells (Figure 10E).^[^ ^145^ ^]^

In addition to mimicking the functions of biomolecules in the wound microenvironment, regulating the wound microenvironment also includes ameliorating the oxidative and inflammatory microenvironments (e.g., reducing the levels of ROS and inflammatory factors in chronic wounds). However, few peptide hydrogel materials are designed for these regulatory roles. In the future, we believe that peptide‐based hydrogels will be widely explored to mimic the biological functions of ECM and regulate the wound microenvironment to treat wound tissue with spatiotemporal control.

## 6 Conclusions and Future Perspectives

Wound healing, especially for chronic wounds, is a complicated process with numerous steps, creating difficulties for developing treatment approaches for chronic wounds. Molecular self‐assembly has become a powerful tool for constructing hydrogels, which are finding increasing applications in biomedicine. Peptide‐based self‐assembling hydrogels are inherently attractive due to their various advantages.

This review discusses various types of peptide‐based self‐assembling hydrogels and their corresponding wound healing applications, providing some application‐driven design strategies for peptide‐based self‐assembly. We discuss the current peptide self‐assembling systems that rely on different interactions and describe the advantages of peptide‐based self‐assembling hydrogels compared to other agents for wound treatment. In addition to their excellent biocompatibility, biodegradability, and easy functionalization, peptide‐based self‐assembling hydrogels also show the ability of ligand‐receptor recognition, stimulus‐responsive self‐assembly, and the ability to mimic the natural ECM. The assembly process and geometric structure of peptide‐based self‐assembling materials can be regulated by the physiological pH, temperature, ionic strength, and enzymes. Based on these features, a range of peptide‐based self‐assembling materials have been developed as wound dressings to improve therapeutic approaches and speed wound healing.

This review highlights the recent advances in self‐assembling peptides and peptide‐based materials for different wound healing processes, including hemostasis, infection and inflammation response, proliferation, and remodeling. Meanwhile, peptide‐based hydrogels can provide temporal control for long‐term wound treatment, sustained drug release, and controllable degradation. These hydrogels also provide excellent spatial control over wound healing as follows: The soft hydrogel fits the edges of wound tissues; the nanofibrous structure mimics the natural ECM; and the hydrogel scaffold regulates the wound microenvironment. Therefore, the personalized design of peptide‐based hydrogels shows considerable potential to provide wound treatment with spatiotemporal control, as embodied by therapeutic peptides with multiple functions and peptides that regulate and remodel the wound microenvironment.

Peptidyl self‐assembling hydrogels have shown excellent treatment effects in various stages of wound healing and represent a promising emerging research frontier. A potential research direction is the design of self‐assembling peptide‐based hydrogels that combine with or wrap natural pro‐healing peptides (e.g., OA‐GL12 and RL‐QN15) or peptide growth factors (e.g., EGF and FGF) to i) improve the healing ability of natural healing peptides; ii) promote the assembly of peptides into hydrogels; and iii) anchor bioactive molecules in the application area to maintain the continuous release of drugs and improve their therapeutic effects. For instance, when self‐assembling peptide fiber scaffolds were combined with EGF or FGF, growth factors were continuously released from the hydrogel, accelerating the speed of wound healing.^[^ ^146^ ^]^

Advanced materials that interact with the immune system in a spatiotemporally controlled manner are receiving increasing attention,^[^ ^24^, ^147^ ^]^ and regulating the immune system is also conducive to the healing of chronic wounds.^[^ ^148^ ^]^ Recently, several studies have reported hydrogel dressings with immunomodulation functions in wounds. These functions include: i) Scavenging overexpressed inflammatory chemokines;^[^ ^21b^ ^]^ ii) modulating macrophage polarization (from M1 to M2);^[^ ^149^ ^]^ and iii) releasing bioactive components such as cytokines, growth factors, and genes.^[^ ^24b^ ^]^ However, the research and application of peptide‐based self‐assembling hydrogels that participate in the immune regulation of wounds are lacking, and the ability of these materials to provide spatiotemporal control over immune response requires further exploration.

Although numerous types of self‐assembling peptide‐based materials have been developed, several key challenges remain: i) The physical and chemical properties of peptide‐based hydrogels require further optimization in terms of tissue adhesion, malleability, and degradation rate; ii) the best suture and implantation methods should be identified to promote wound healing; iii) further research is needed on peptide‐based hydrogels encapsulating growth factors and small‐molecule drugs to enable the release of drugs in a dynamic and controlled manner; iv) few studies have been conducted on diabetic wound treatment, and more peptide‐based hydrogels for treating diabetic wounds need to be developed; v) the mechanism of the hydrogel in promoting wound repair is still stay in the superficial analysis of promoting the wound healing process, and there is a lack of in‐depth mechanistic exploration; and vi) the application of peptide‐based hydrogels in scarless tissue repair has not been explored and is deserving of more attention.

In general, various factors should be considered in the design of self‐assembling peptide‐based hydrogels for different applications, including their effectiveness and practicality. More personalized designs are also needed; for instance, peptidyl or protein‐based hydrogels could be designed according to different ECMs found in different wound tissues. Future research will also likely focus on multifunctional and multi‐component biomaterials along with 3D printing technology and wound monitoring equipment. These tools are expected to aid in the design of more portable next‐generation wound‐healing hydrogel materials. Although the clinical and translational application of these materials requires additional in vivo investigation, we hope that peptidyl self‐assemblies will be successfully applied to treat wounds in the future and further advance the wound healing field.

## Conflict of Interest

The authors declare no conflict of interest.
